# Supplementary material for: Clonal diversification and histogenesis of malignant germ cell tumours
Source: Nat Commun. 2022 Aug 11;13:4272. doi: 10.1038/s41467-022-31375-4 (PMC9372159; doi:10.1038/s41467-022-31375-4)
Supplement: Supplementary file 1 — Supplementary information [file 41467_2022_31375_MOESM1_ESM.pdf]

SUPPLEMENTARY FIGURES

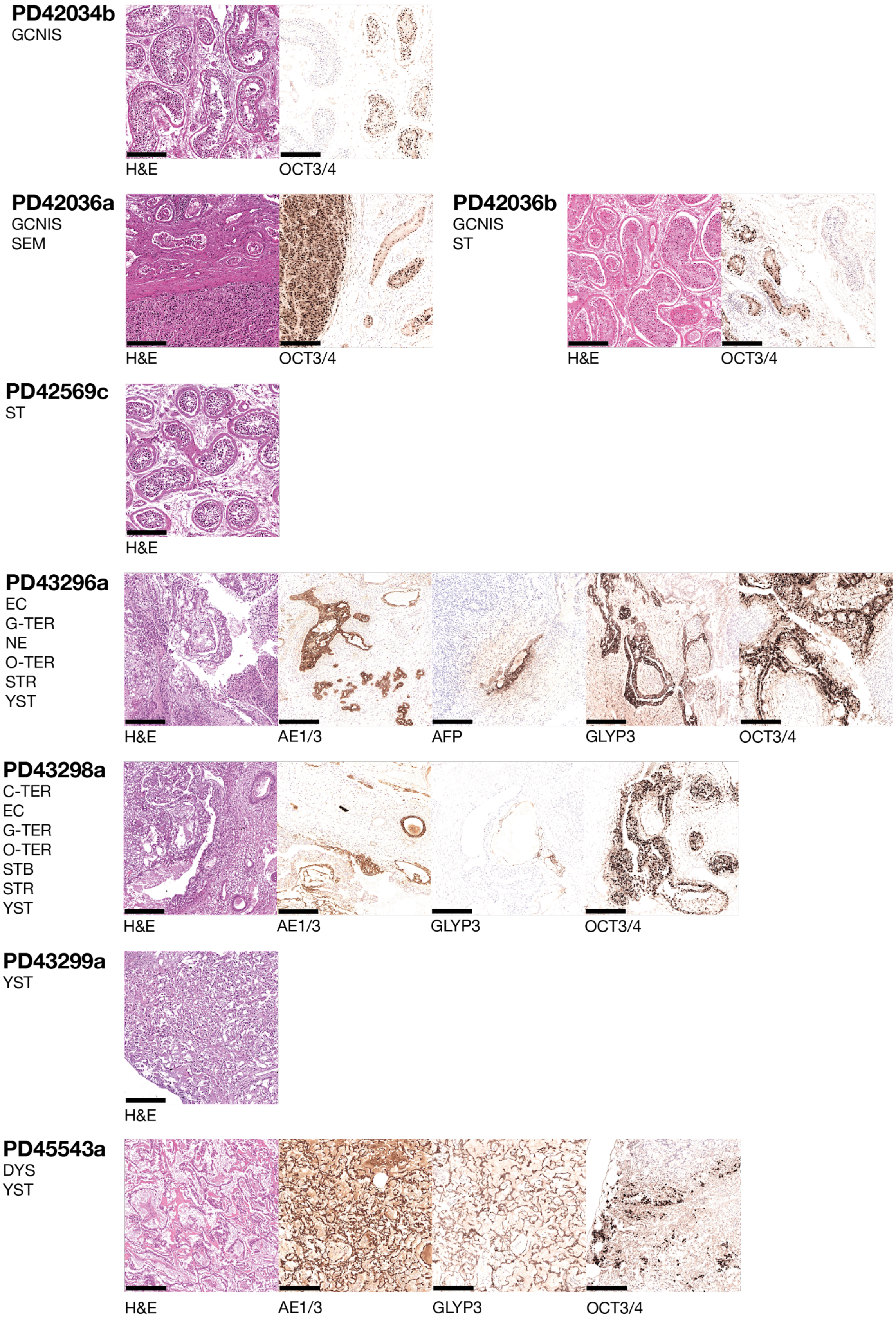

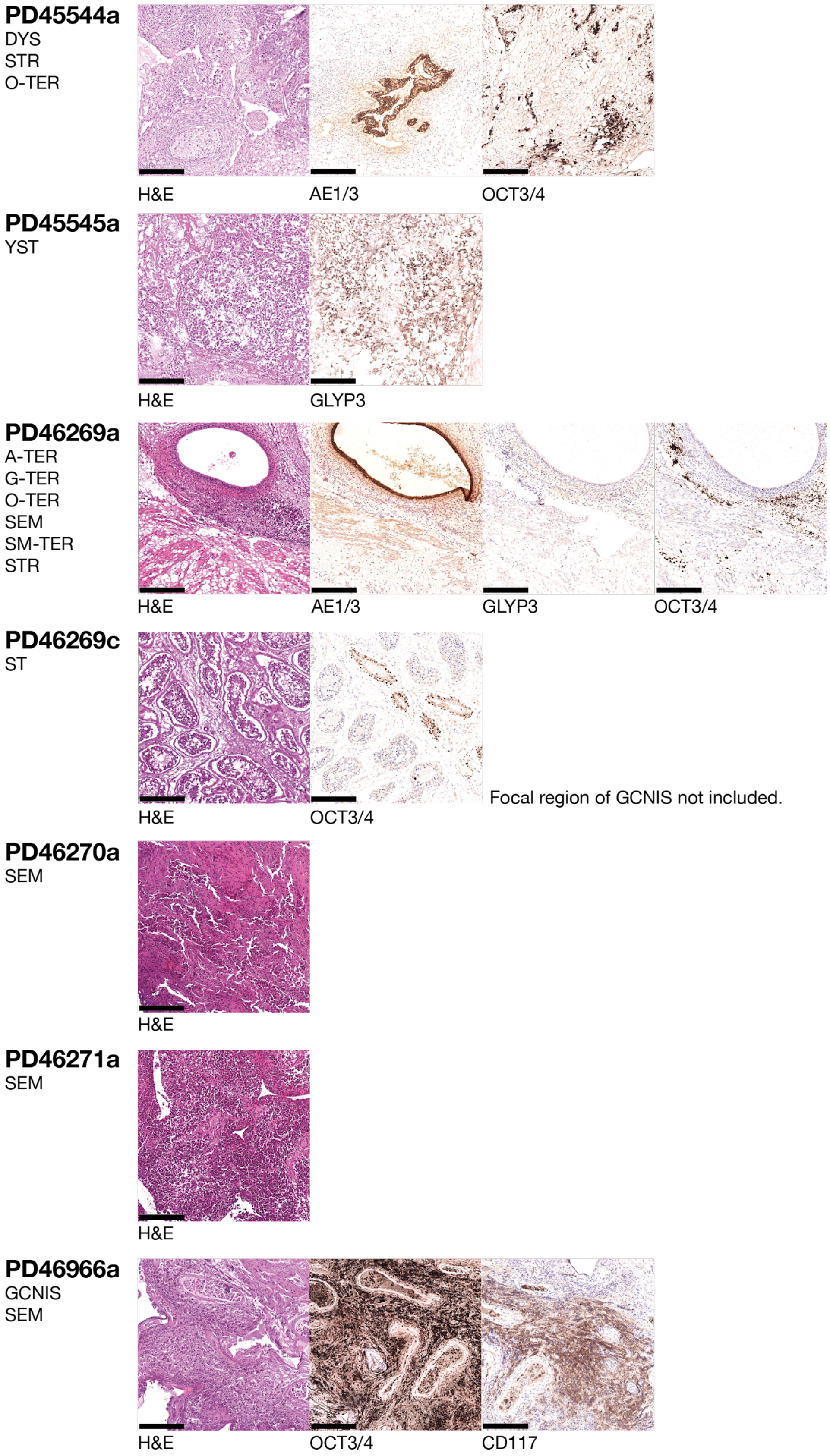
PD45544a
DYS
STR
O-TER
H&E
AE1/3
OCT3/4
PD45545a
YST
H&E
GLYP3
PD46269a
A-TER
G-TER
O-TER
SEM
SM-TER
STR
H&E
AE1/3
GLYP3
OCT3/4
PD46269c
ST
H&E
OCT3/4
Focal region of GCNIS not included.
PD46270a
SEM
H&E
PD46271a
SEM
H&E
PD46966a
GCNIS
SEM
H&E
OCT3/4
CD117

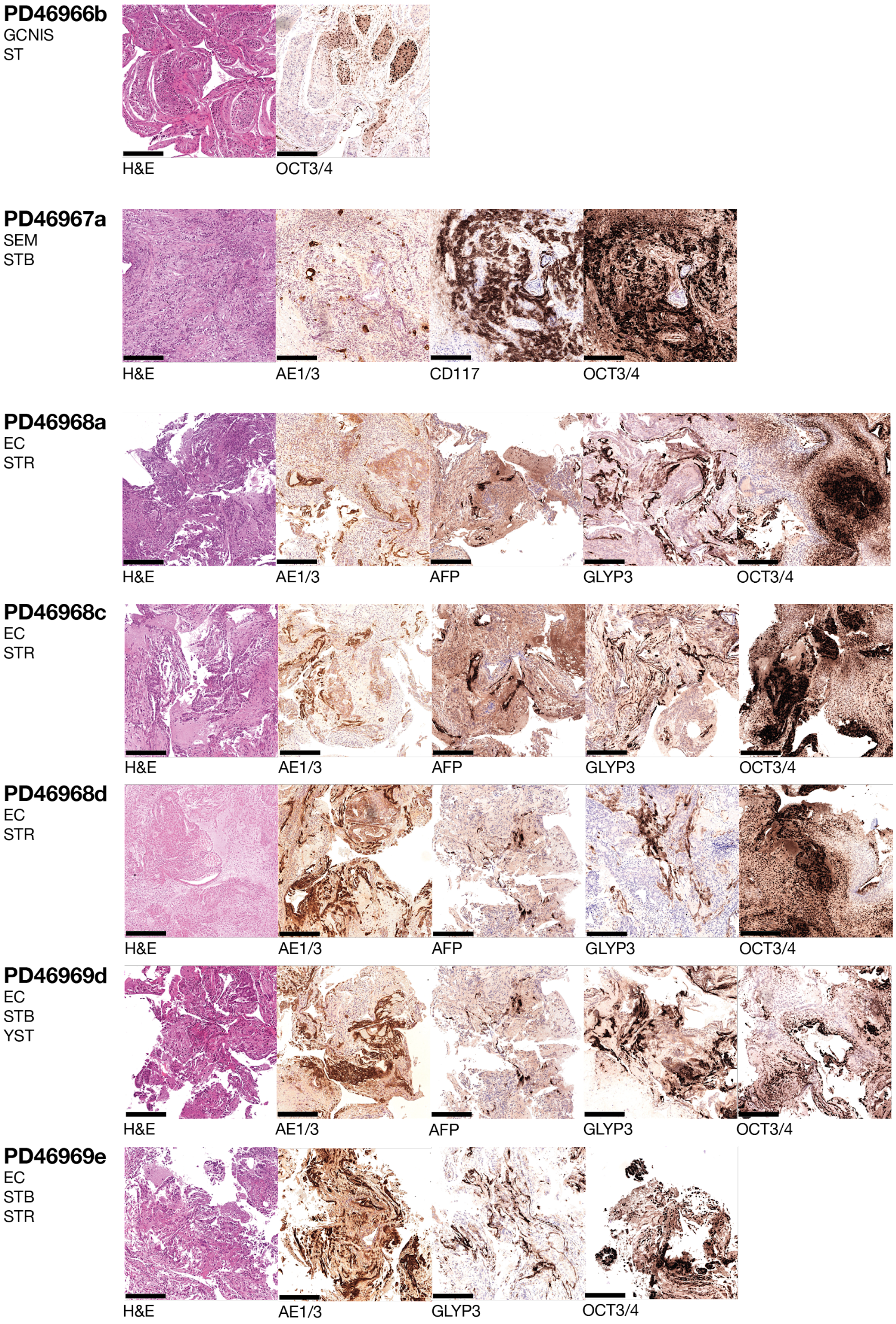
PD46966b
GCNIS
ST
H&E
OCT3/4
PD46967a
SEM
STB
H&E
AE1/3
CD117
OCT3/4
PD46968a
EC
STR
H&E
AE1/3
AFP
GLYP3
OCT3/4
PD46968c
EC
STR
H&E
AE1/3
AFP
GLYP3
OCT3/4
PD46968d
EC
STR
H&E
AE1/3
AFP
GLYP3
OCT3/4
PD46969d
EC
STB
YST
H&E
AE1/3
AFP
GLYP3
OCT3/4
PD46969e
EC
STB
STR
H&E
AE1/3
GLYP3
OCT3/4

Supplementary Figure 1 | Histological images from each biopsy included in the microdissection cohort. Micrographs taken from a reference H&E slide and any immunohistochemistry that informed histological categorisation. Gamma changes have been applied to these micrographs to help distinguish the histological features. Immunohistochemistry was undertaken to resolve ambiguity in the histological classification of each tissue with the first run of each antibody that provided satisfactory positive and negative controls used for review. Underneath each case ID is a list of the histologies successfully isolated from it. For some cases, DNA and mRNA libraries were not successfully generated from all components found on the reference slides. A-TER, adipose teratoma; C-TER, cartilage teratoma; DYS, dysgerminoma; EC, embryonal carcinoma; GCNIS, germ cell neoplasia in situ; G-TER, mature glandular teratoma, NE, neuroepithelium; O-TER, other epithelial teratoma; SM-TER, smooth muscle teratoma; SEM, seminoma; STB, syncytiotrophoblasts; STR, malignant stroma; ST, healthy seminiferous tubules; YST, yolk sac tumour. Scale bars denote 250 microns.

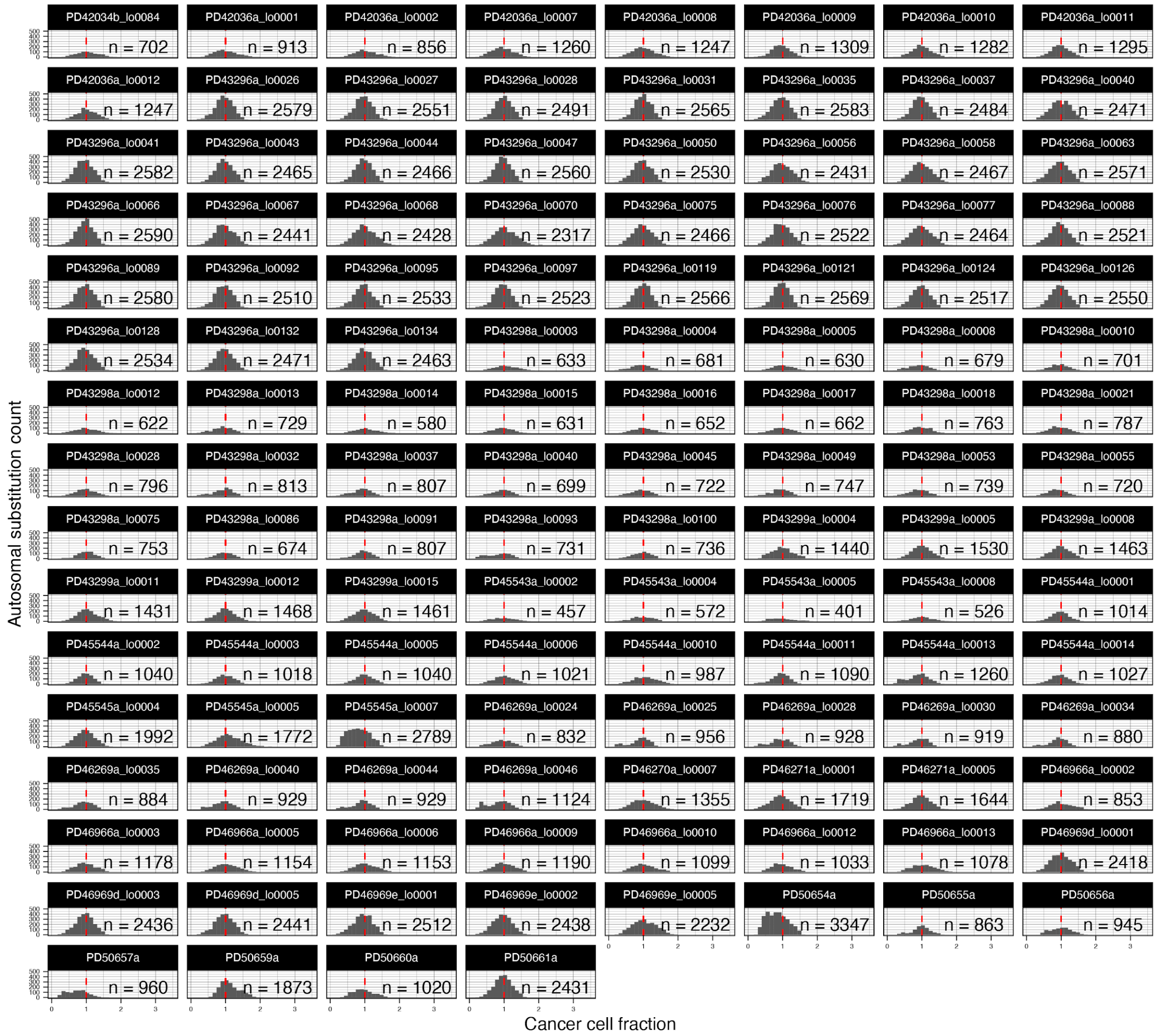

Supplementary Figure 2 | Histograms of the cancer cell fraction for substitutions lying on the autosomal genome in each GCT sample. Only samples with an estimated minimum of five reads per chromosome copy were included, reflecting the threshold used to have sufficient confidence to include the associated copy number data in downstream analyses. The number of substitutions underlying each distribution is stated on each plot.

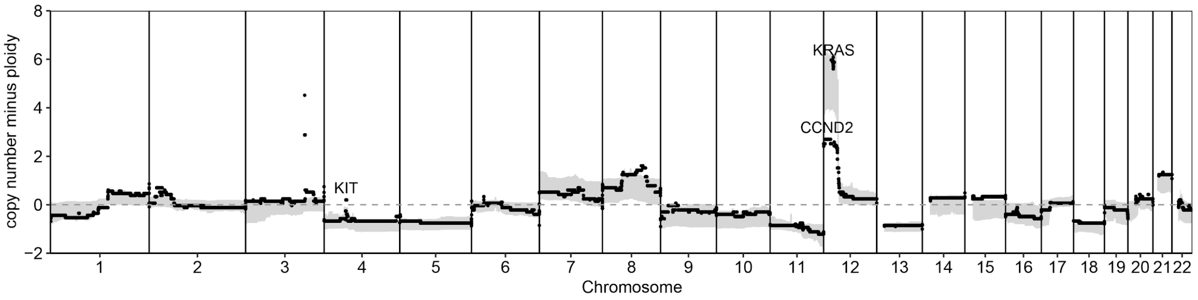

Supplementary Figure 3 | Aggregated copy number across the microdissection cohort compared to TCGA reference data [6]. The study cohort average excludes the single prepubertal case (PD43299) as none such cases were present in the TCGA dataset. The black line denotes the study mean (n = 11 invasive, postpubertal GCTs) with the grey band representing the interquartile range of the eligible TCGA samples (n = 103 tumours).

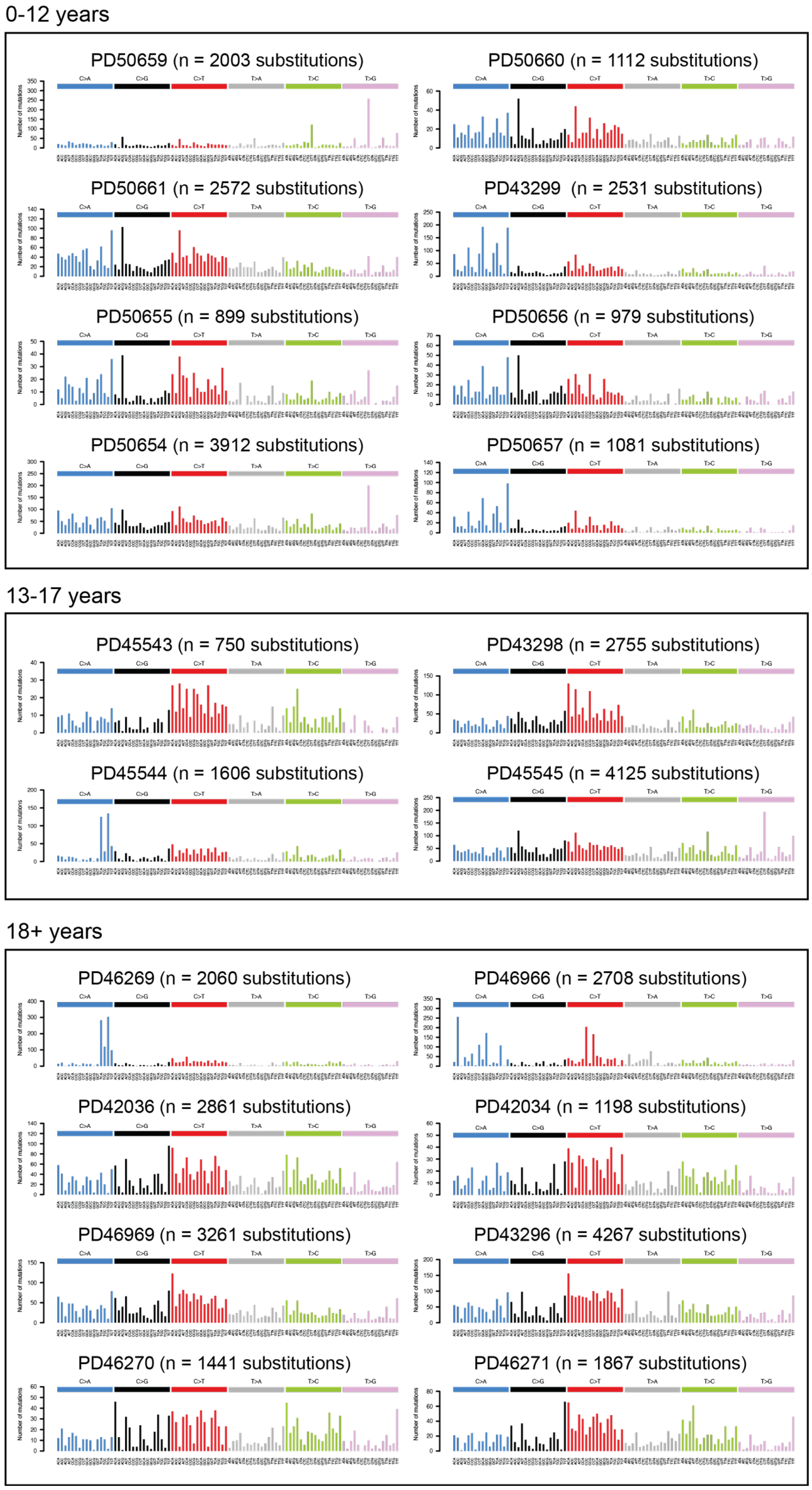

Supplementary Figure 4 | Trinucleotide context plots of all unique substitutions per GCT. Each GCT is arranged by age.

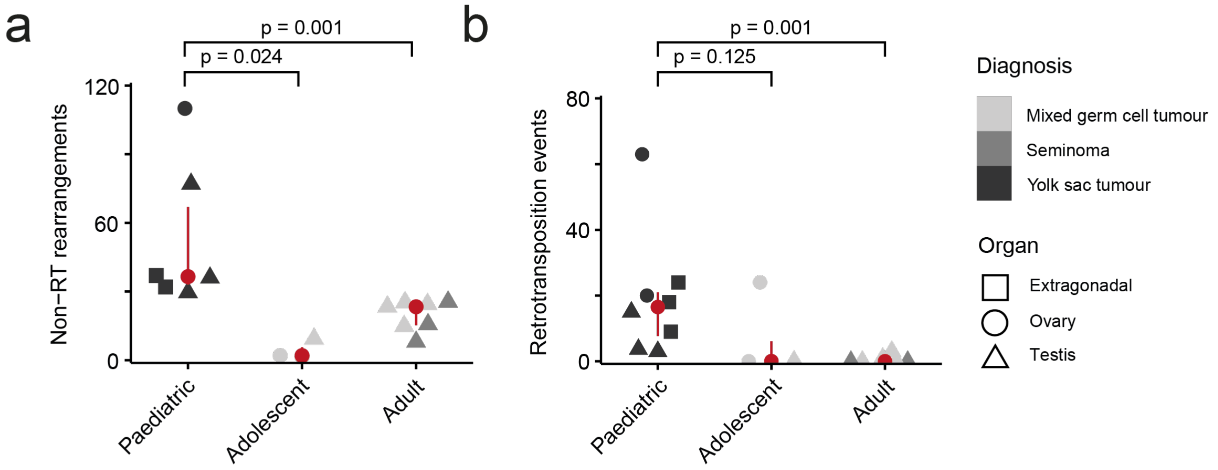

Supplementary Figure 5 | Structural variant burden per invasive GCT. (a) Mean burden of all structural variants except retrotransposition (RT) events per tumour. Samples which have undergone a chromothripsis-like event are excluded. Cases are binned by age; Paediatric (0 - 12 years, n = 6 tumours), Adolescent (13 - 17 years, n = 3 tumours) and Adult (18+ years, n = 7 tumours). Each red dot represents the median rearrangement count and the lines are the interquartile range. P-values were acquired using the two-sided Wilcoxon rank-sum test (Paediatric vs Adolescent, p = 0.024; Paediatric vs adult, p = 0.001). (b) Mean retrotransposition events called per tumour, using the same age bins and point ranges as shown in (a); Paediatric (n = 8 tumours), Adolescent (n = 4 tumours) and Adult (n = 7 tumours). P values were acquired using the two-sided Wilcoxon rank-sum test (Paediatric vs Adolescent, p = 0.125; Paediatric vs Adult, p = 0.001). No adjustments are made for multiple comparisons in (a) or (b) and in situ disease is excluded from the analysis.

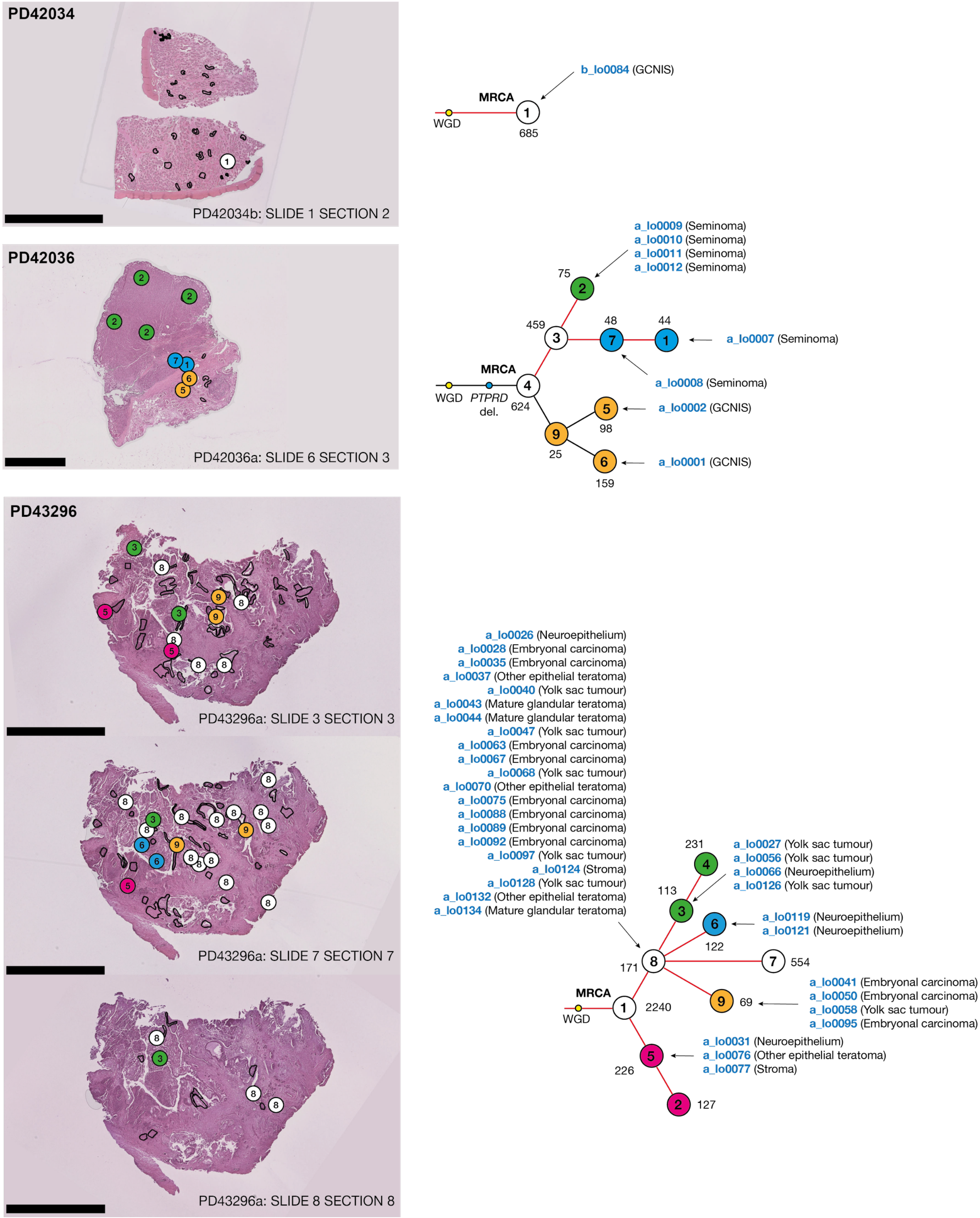
PD42034
PD42034b: SLIDE 1 SECTION 2
b_lo0084 (GCNIS)
MRCA
WGD
685
PD42036
PD42036a: SLIDE 6 SECTION 3
a_lo0009 (Seminoma)
a_lo0010 (Seminoma)
a_lo0011 (Seminoma)
a_lo0012 (Seminoma)
75
459
48
44
a_lo0007 (Seminoma)
MRCA
a_lo0008 (Seminoma)
WGD
PTPRD
del.
624
a_lo0002 (GCNIS)
98
25
a_lo0001 (GCNIS)
159
PD43296
PD43296a: SLIDE 3 SECTION 3
PD43296a: SLIDE 7 SECTION 7
PD43296a: SLIDE 8 SECTION 8
a_lo0026 (Neuroepithelium)
a_lo0028 (Embryonal carcinoma)
a_lo0035 (Embryonal carcinoma)
a_lo0037 (Other epithelial teratoma)
a_lo0040 (Yolk sac tumour)
a_lo0043 (Mature glandular teratoma)
a_lo0044 (Mature glandular teratoma)
a_lo0047 (Yolk sac tumour)
a_lo0063 (Embryonal carcinoma)
a_lo0067 (Embryonal carcinoma)
a_lo0068 (Yolk sac tumour)
a_lo0070 (Other epithelial teratoma)
a_lo0075 (Embryonal carcinoma)
a_lo0088 (Embryonal carcinoma)
a_lo0089 (Embryonal carcinoma)
a_lo0092 (Embryonal carcinoma)
a_lo0097 (Yolk sac tumour)
a_lo0124 (Stroma)
a_lo0128 (Yolk sac tumour)
a_lo0132 (Other epithelial teratoma)
a_lo0134 (Mature glandular teratoma)
231
a_lo0027 (Yolk sac tumour)
a_lo0056 (Yolk sac tumour)
a_lo0066 (Neuroepithelium)
a_lo0126 (Yolk sac tumour)
113
a_lo0119 (Neuroepithelium)
a_lo0121 (Neuroepithelium)
122
171
554
MRCA
2240
WGD
69
a_lo0041 (Embryonal carcinoma)
a_lo0050 (Embryonal carcinoma)
a_lo0058 (Yolk sac tumour)
a_lo0095 (Embryonal carcinoma)
a_lo0031 (Neuroepithelium)
a_lo0076 (Other epithelial teratoma)
a_lo0077 (Stroma)
226
127

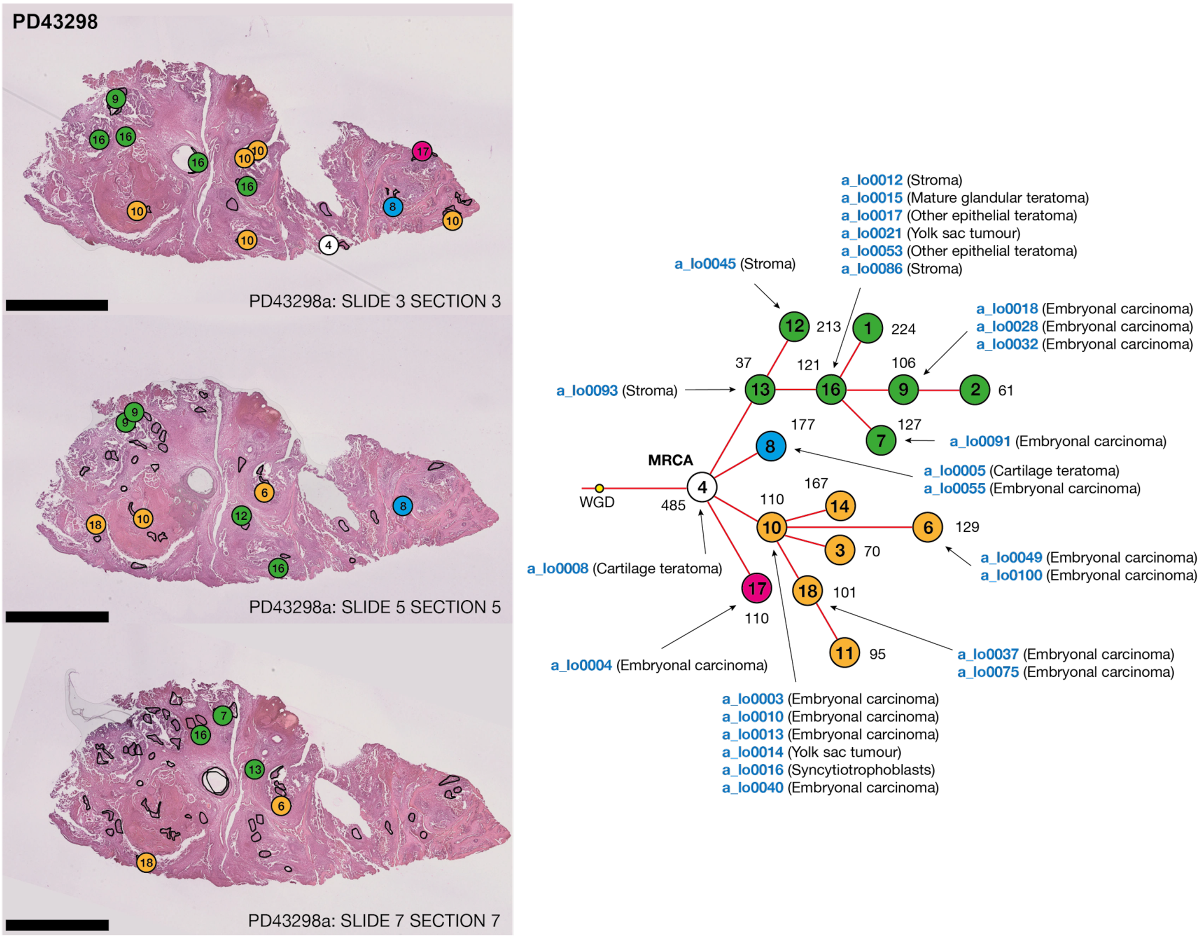
PD43298
PD43298a: SLIDE 3 SECTION 3
PD43298a: SLIDE 5 SECTION 5
PD43298a: SLIDE 7 SECTION 7
a_lo0012 (Stroma)
a_lo0015 (Mature glandular teratoma)
a_lo0017 (Other epithelial teratoma)
a_lo0021 (Yolk sac tumour)
a_lo0053 (Other epithelial teratoma)
a_lo0086 (Stroma)
a_lo0045 (Stroma)
a_lo0018 (Embryonal carcinoma)
a_lo0028 (Embryonal carcinoma)
a_lo0032 (Embryonal carcinoma)
a_lo0093 (Stroma)
a_lo0091 (Embryonal carcinoma)
MRCA
WGD
a_lo0005 (Cartilage teratoma)
a_lo0055 (Embryonal carcinoma)
a_lo0049 (Embryonal carcinoma)
a_lo0100 (Embryonal carcinoma)
a_lo0008 (Cartilage teratoma)
a_lo0037 (Embryonal carcinoma)
a_lo0075 (Embryonal carcinoma)
a_lo0004 (Embryonal carcinoma)
a_lo0003 (Embryonal carcinoma)
a_lo0010 (Embryonal carcinoma)
a_lo0013 (Embryonal carcinoma)
a_lo0014 (Yolk sac tumour)
a_lo0016 (Syncytiotrophoblasts)
a_lo0040 (Embryonal carcinoma)

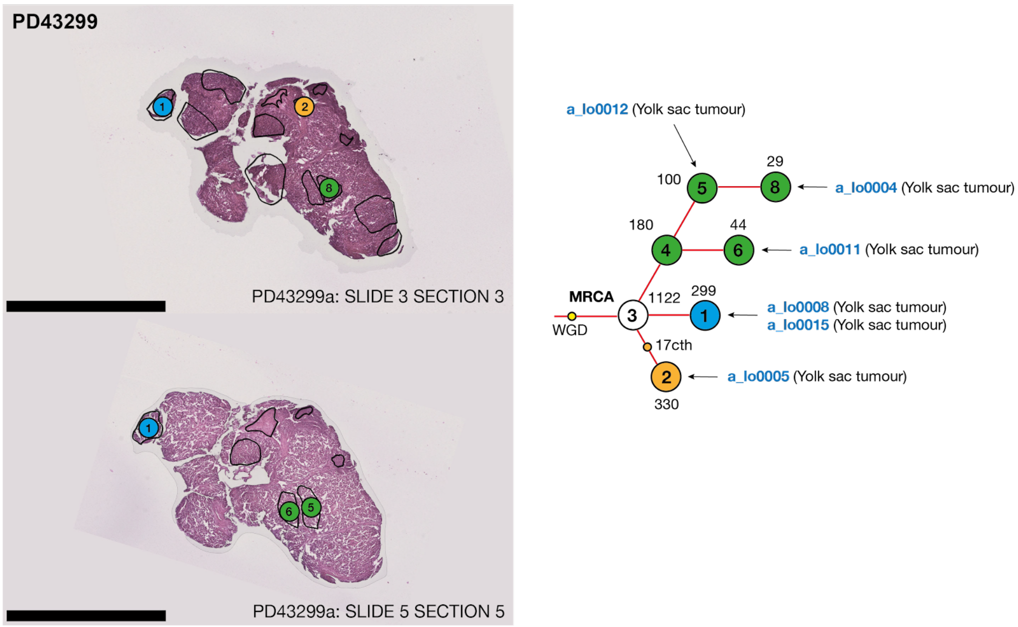
PD43299
PD43299a: SLIDE 3 SECTION 3
PD43299a: SLIDE 5 SECTION 5
a_lo0012 (Yolk sac tumour)
a_lo0004 (Yolk sac tumour)
a_lo0011 (Yolk sac tumour)
MRCA
WGD
17cth
a_lo0008 (Yolk sac tumour)
a_lo0015 (Yolk sac tumour)
a_lo0005 (Yolk sac tumour)

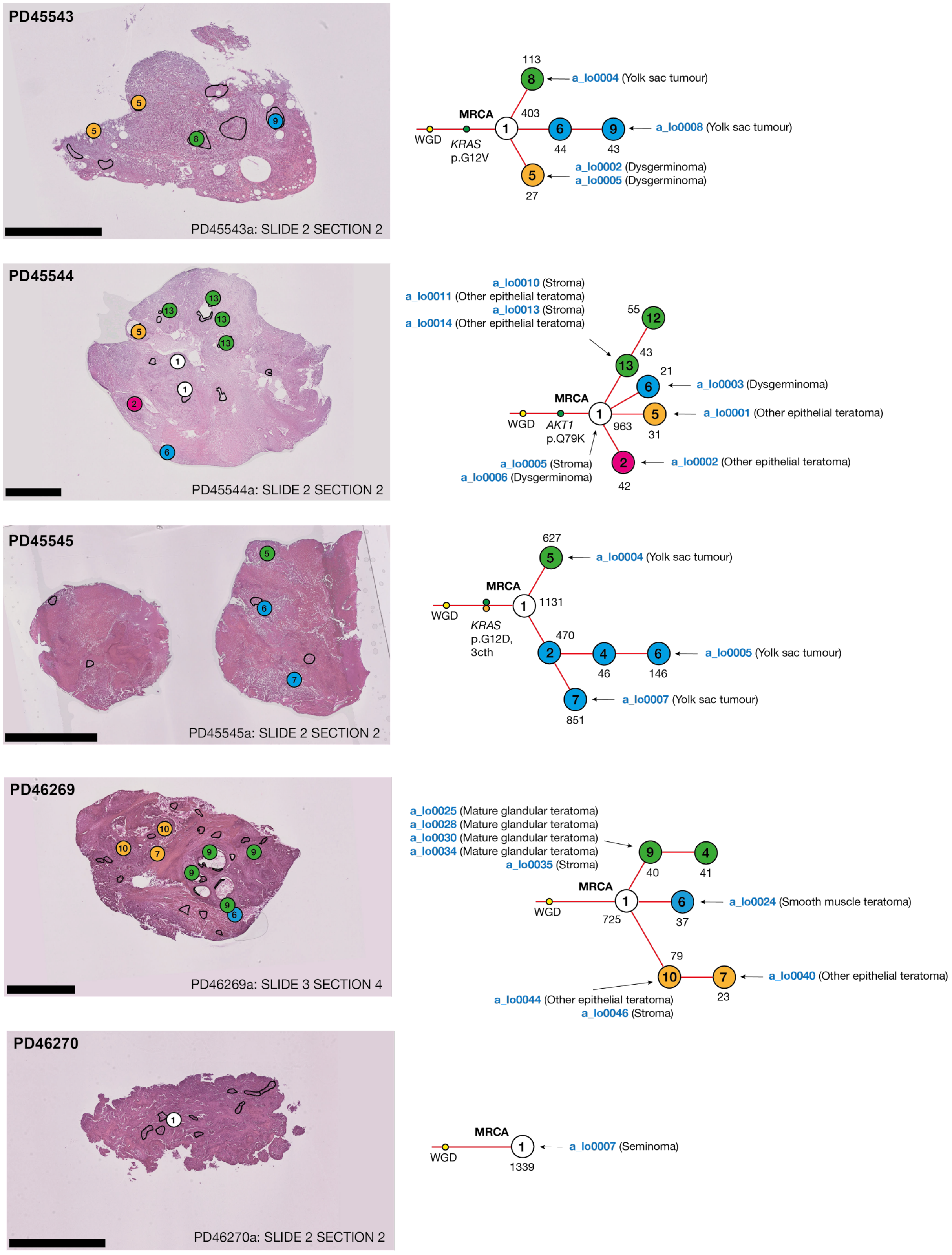

PD45543
PD45543a: SLIDE 2 SECTION 2
113
403
44
43
27
MRCA
WGD
KRAS
p.G12V
a_lo0004 (Yolk sac tumour)
a_lo0008 (Yolk sac tumour)
a_lo0002 (Dysgerminoma)
a_lo0005 (Dysgerminoma)
PD45544
PD45544a: SLIDE 2 SECTION 2
a_lo0010 (Stroma)
a_lo0011 (Other epithelial teratoma)
a_lo0013 (Stroma)
a_lo0014 (Other epithelial teratoma)
55
43
21
963
31
42
MRCA
WGD
AKT1
p.Q79K
a_lo0003 (Dysgerminoma)
a_lo0001 (Other epithelial teratoma)
a_lo0002 (Other epithelial teratoma)
a_lo0005 (Stroma)
a_lo0006 (Dysgerminoma)
PD45545
PD45545a: SLIDE 2 SECTION 2
627
1131
470
46
146
851
MRCA
WGD
KRAS
p.G12D,
3cth
a_lo0004 (Yolk sac tumour)
a_lo0005 (Yolk sac tumour)
a_lo0007 (Yolk sac tumour)
PD46269
PD46269a: SLIDE 3 SECTION 4
a_lo0025 (Mature glandular teratoma)
a_lo0028 (Mature glandular teratoma)
a_lo0030 (Mature glandular teratoma)
a_lo0034 (Mature glandular teratoma)
a_lo0035 (Stroma)
40
41
725
37
79
23
MRCA
WGD
a_lo0024 (Smooth muscle teratoma)
a_lo0040 (Other epithelial teratoma)
a_lo0044 (Other epithelial teratoma)
a_lo0046 (Stroma)
PD46270
PD46270a: SLIDE 2 SECTION 2
MRCA
WGD
1339
a_lo0007 (Seminoma)

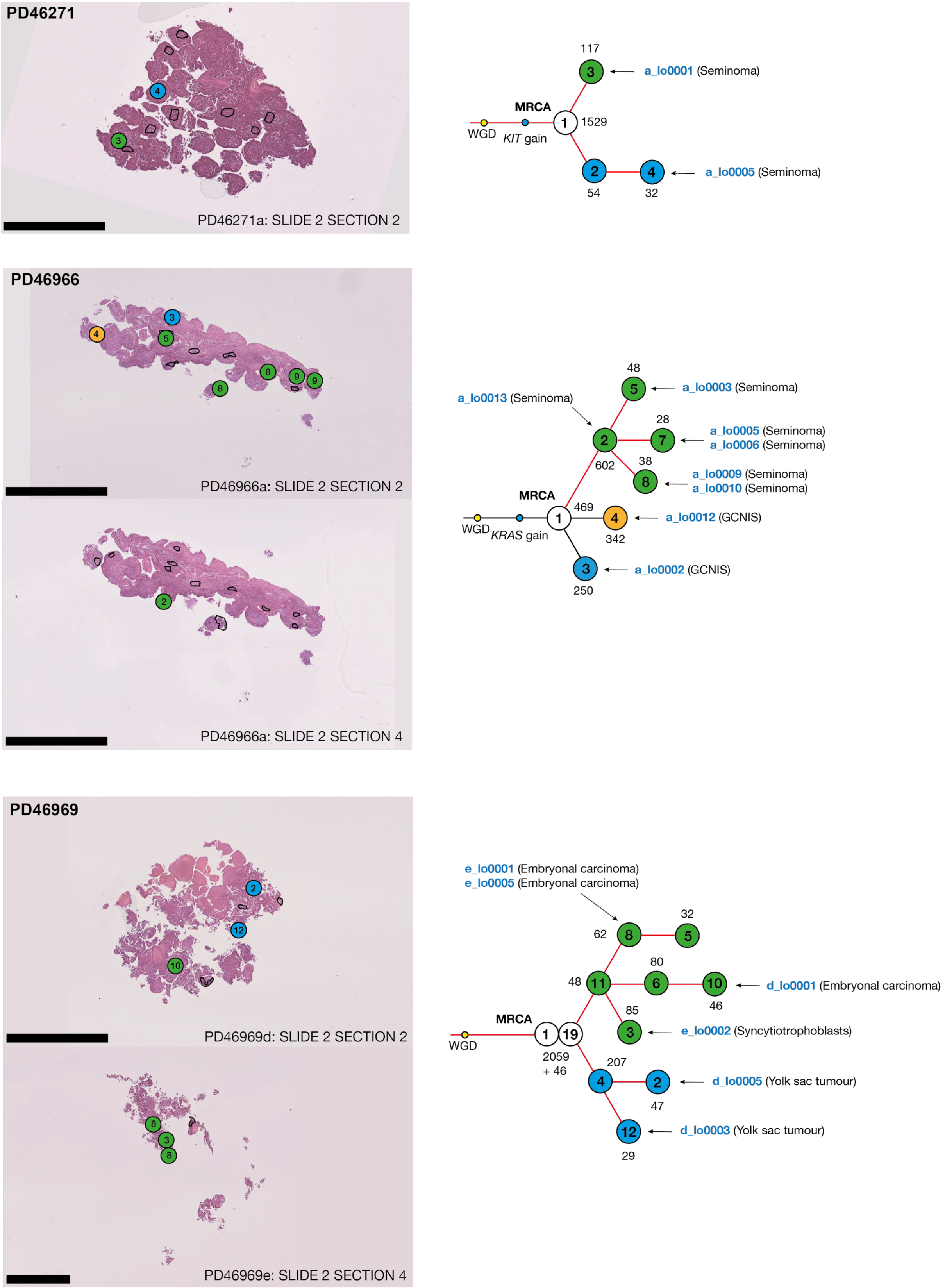

Supplementary Figure 6 | GCT phylogenies mapped back to histological sections. Each circle represents a mutation cluster. The number within the circle is the arbitrary cluster number provided by the phylogeny algorithm. The number adjacent to each circle is the

number of autosomal substitutions supporting it. Black lines between clusters denote an in situ relationship whereas red lines represent linked ancestry following invasion. Each eligible microbiopsy is attributed to the mutation cluster that is the dominant clone (>0.5 cancer cell fraction) found within it. The scale bar for PD42034 indicates 10mm, for PD45545 it is 5mm and for the remainder it is 2.5mm. Gamma changes have been applied to these micrographs to help distinguish the histological features.

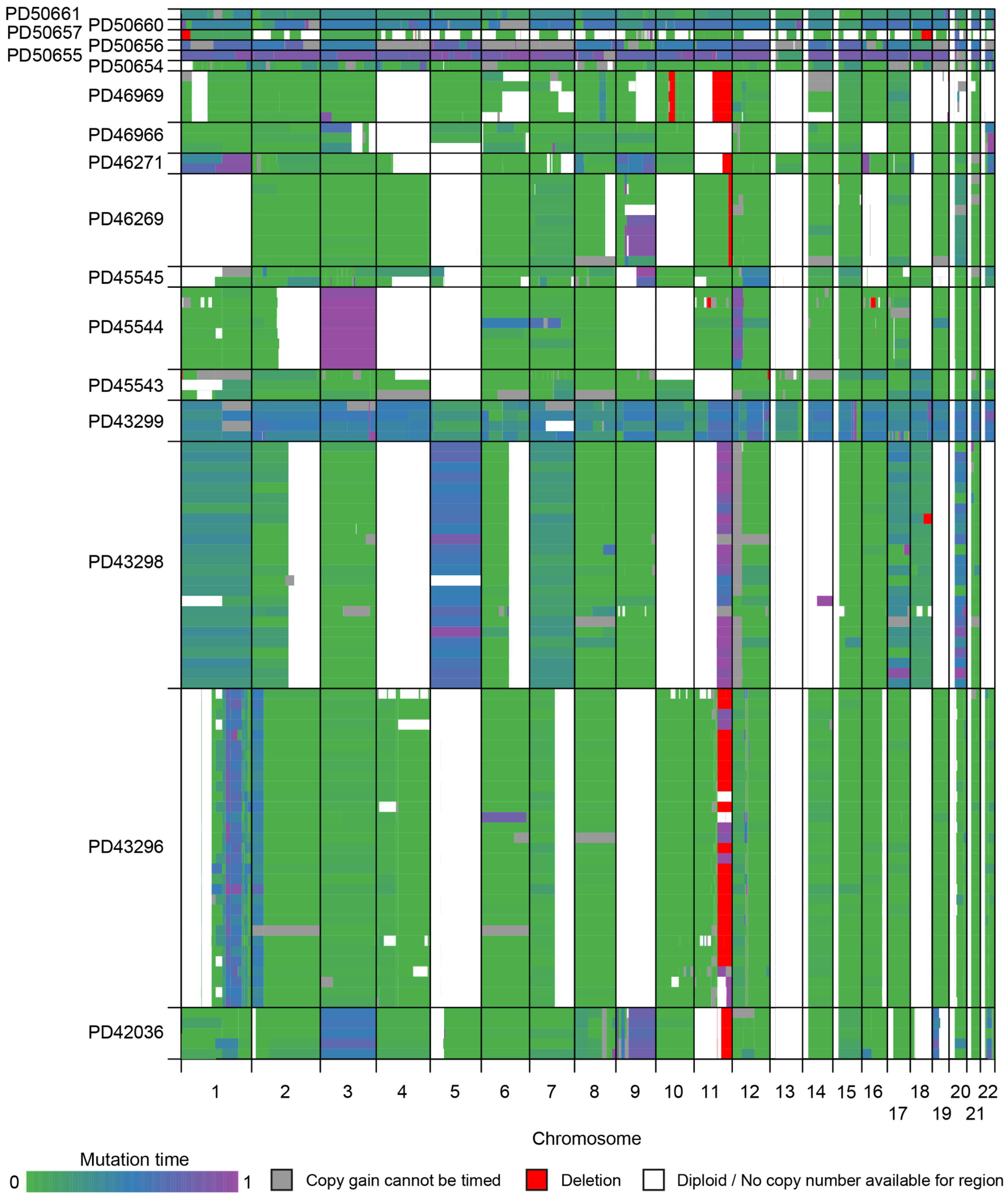

Supplementary Figure 7 | Overview of the mutationtimeR estimates for each copy gain called per GCT sample. Complex, high copy gains and deletions cannot be timed and thus are coloured grey and red respectively. If a segment is gained multiple times and timing estimates are still available, the segment is coloured according to the first gain.

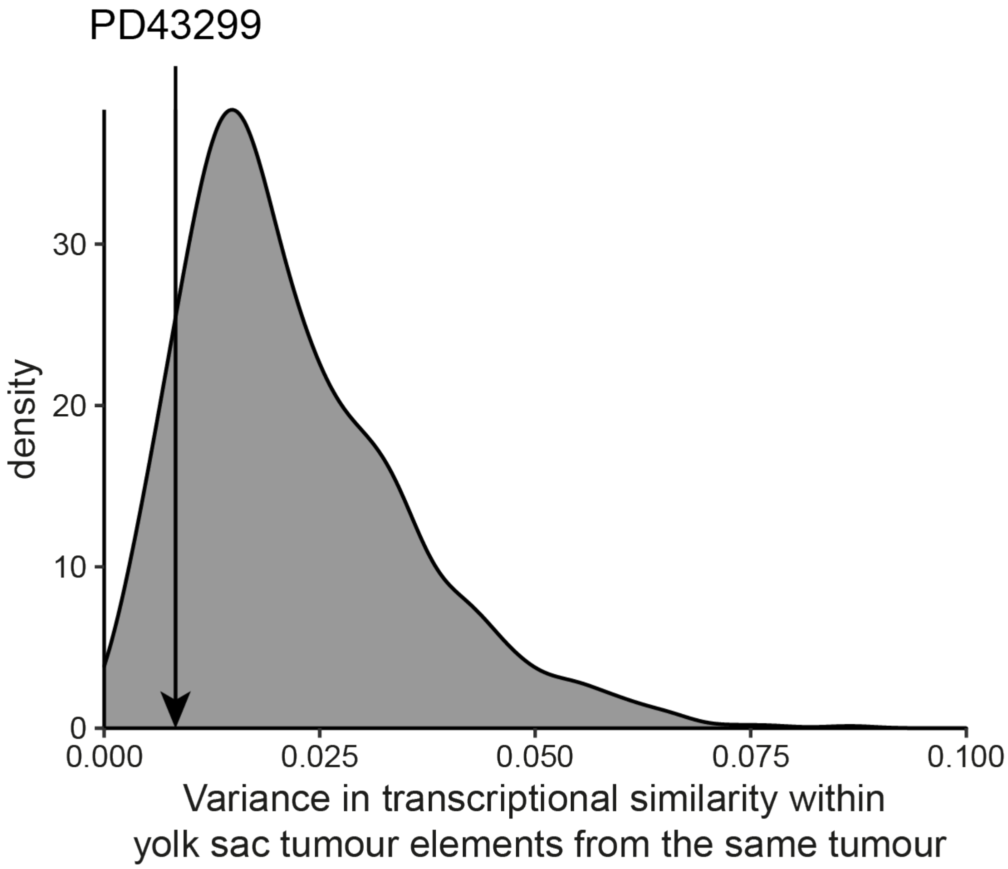

Supplementary Figure 8 | Intra-tumoral yolk sac tumour transcriptional heterogeneity. Pairwise Pearson correlation of the transcriptome of yolk sac tumour elements (n = 31 microdissections) within each tumour (n = 6 tumours) was calculated before the variance of 1,000 random subsets was calculated to achieve the distribution illustrated here. This provides an estimate of the transcriptional variability across the yolk sac tumour within a given tumour. PD43299 did not show a greater transcriptional variability compared to the cohort overall, despite subclonal chromothripsis of chromosome 17.

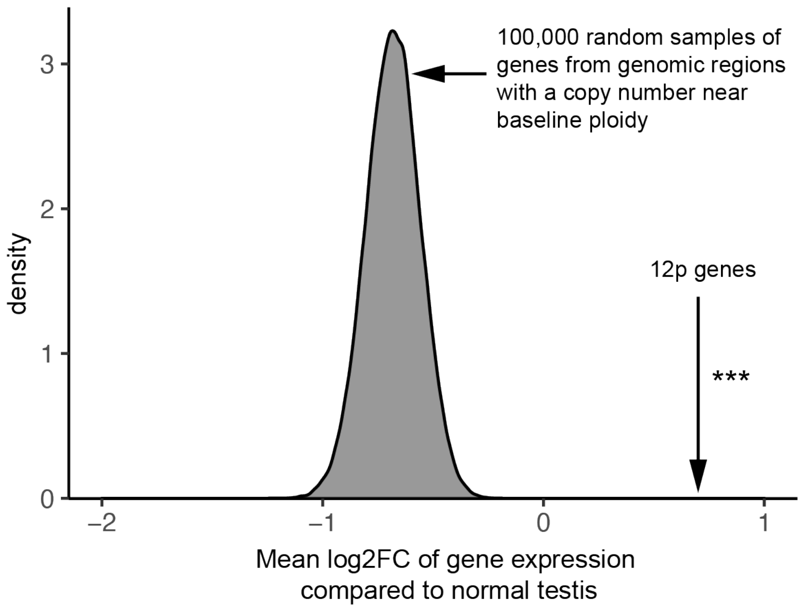

Supplementary Figure 9 | The distribution of the mean log2 fold change in expression compared to normal testis tissues across 100,000 random subsets of genes from GCT genomic regions near baseline ploidy vs 12p genes. No random samples achieved a mean log2 fold change compared to healthy testis tissue higher than that achieved across 12p (one-sided permutation test, $p < 10^{-5}$ indicated by three asterisks). 12,001 autosomal features were adequately expressed across the GCT histologies and/or seminiferous tubules to be included in this analysis. Each random sample contained the same number of genes as those retained on 12p (n = 226).

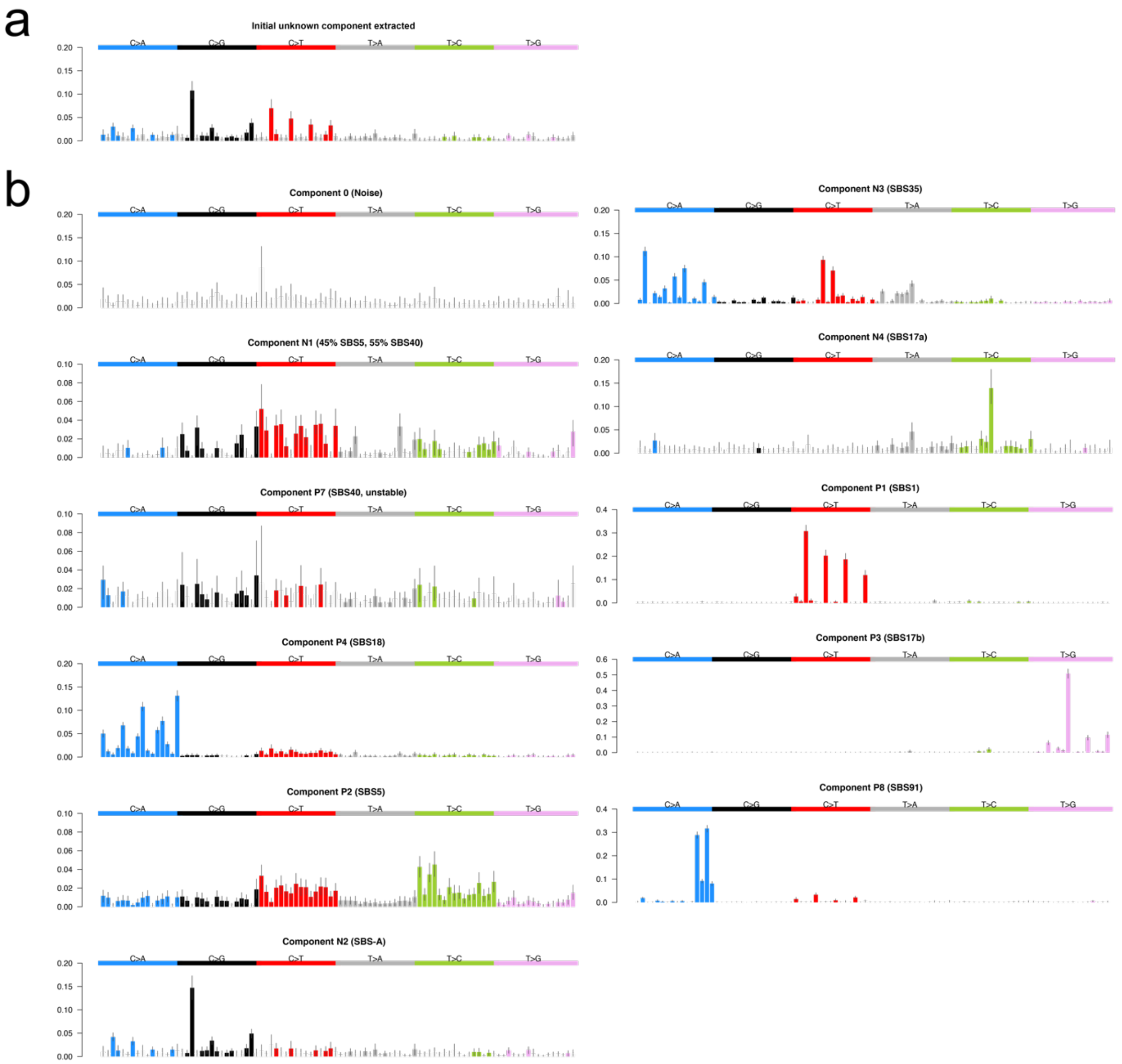

Supplementary Figure 10 | HDP signature extraction components. (a) The initial unknown component extracted using the entire list of COSMIC version 3.2 signatures as a reference. The C>T peaks indicated that it had been extracted with an element of SBS1. (b) The final list of components extracted and the signatures they corresponded to. The whiskers on each bar represent the 95% credible interval for the proposed contribution of that trinucleotide context to the component.

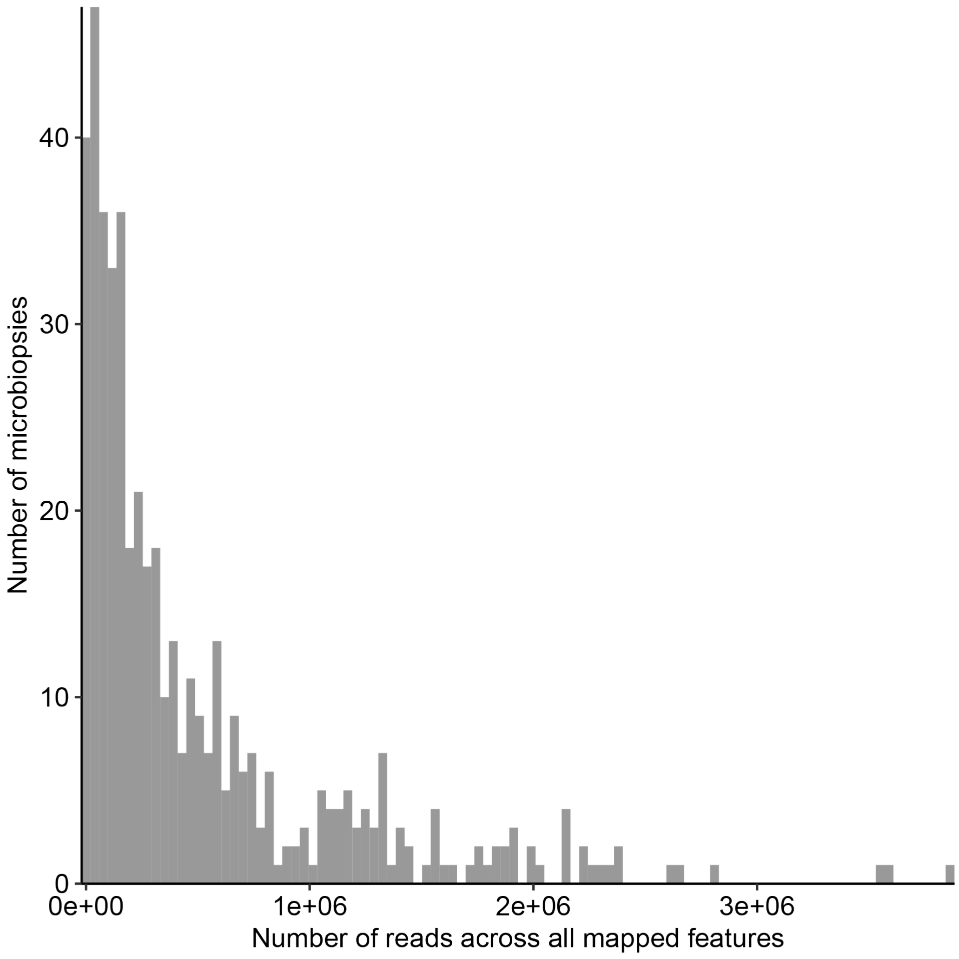

Supplementary Figure 11 | Distribution of the pre-QC total read depth for all microbiopsy transcriptomes. All microbiopsies prior to filtering on the depth and number of features expressed (n = 460 microbiopsies).

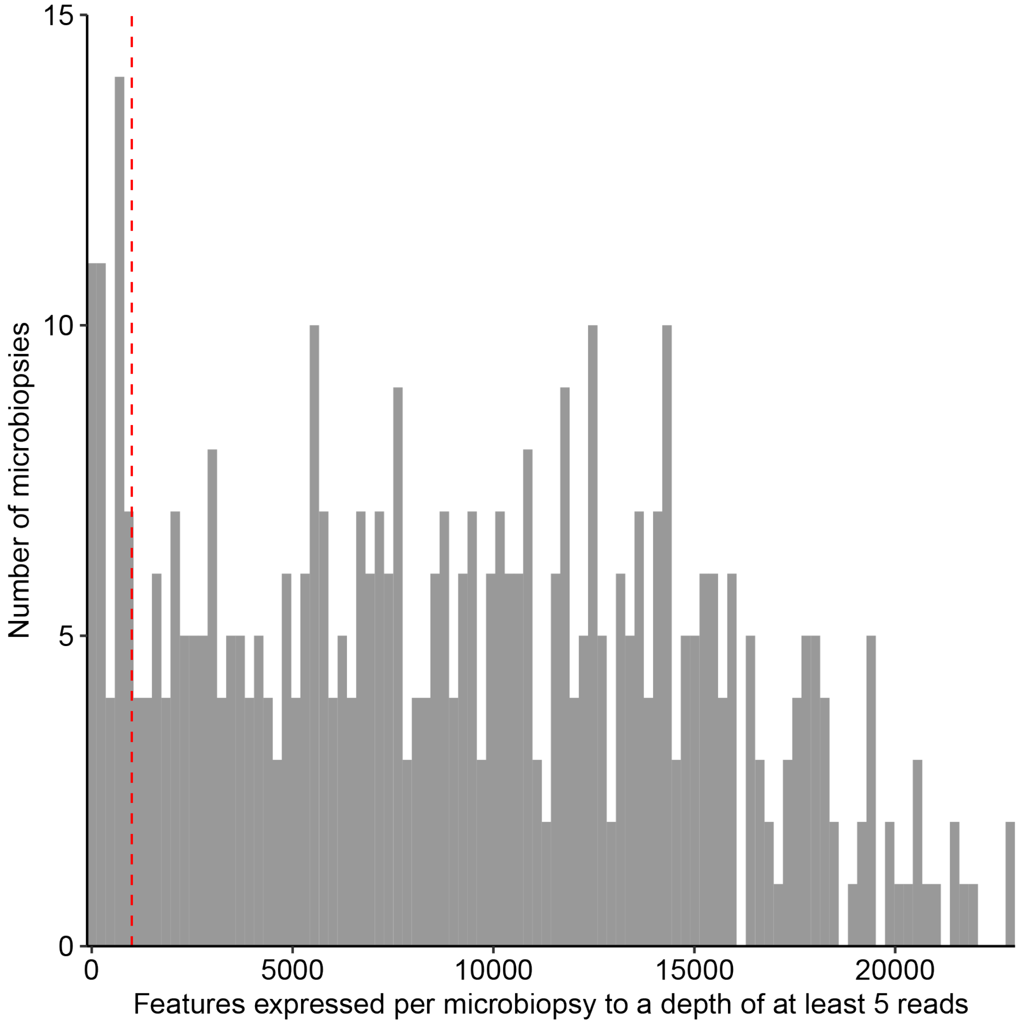

Supplementary Figure 12 | Distribution of the number of genes expressed to a depth of at least 5 reads for all pre-filtered transcriptomes. The dashed red line is drawn at 1,000 genes, the threshold applied to remove low quality samples (416 out of 460 microbiopsies pass this threshold).

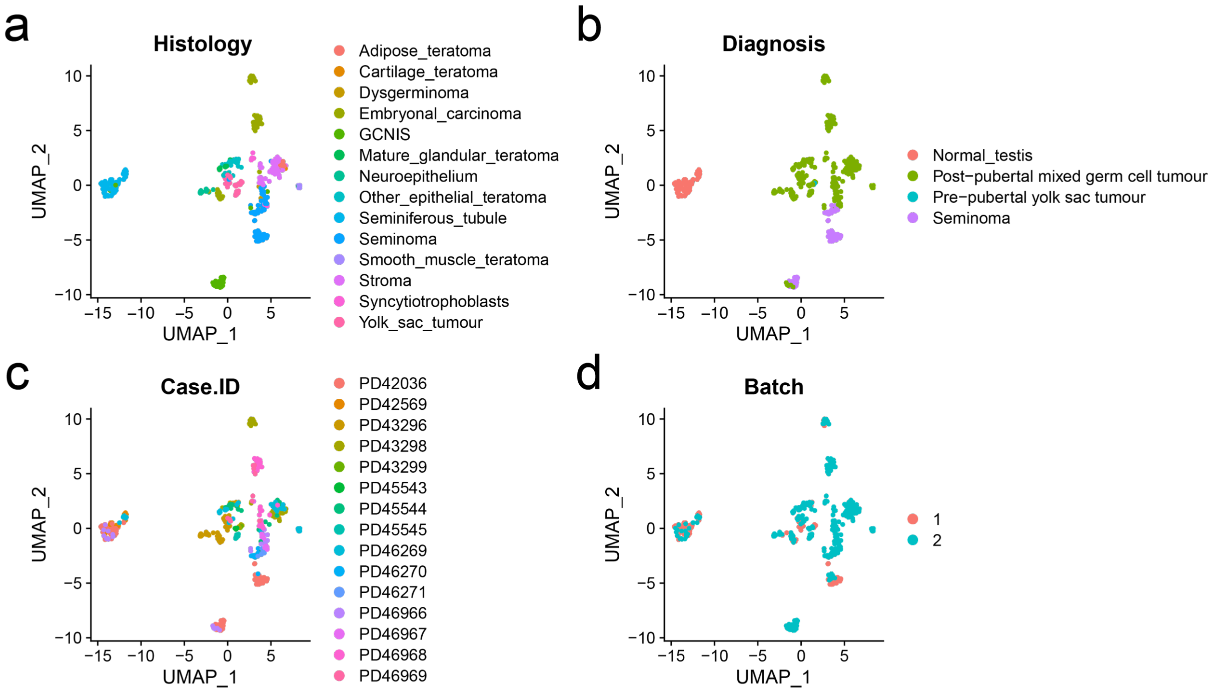

Supplementary Figure 13 | UMAP clustering of RNA microbiopsy data. Coloured by histology (a), tumour diagnosis (b), case ID (c) and batch the samples were submitted in (d). The first 30 dimensions were chosen during the creation of the UMAP. The UMAP encompasses the transcriptomes of 416 microbiopsies.

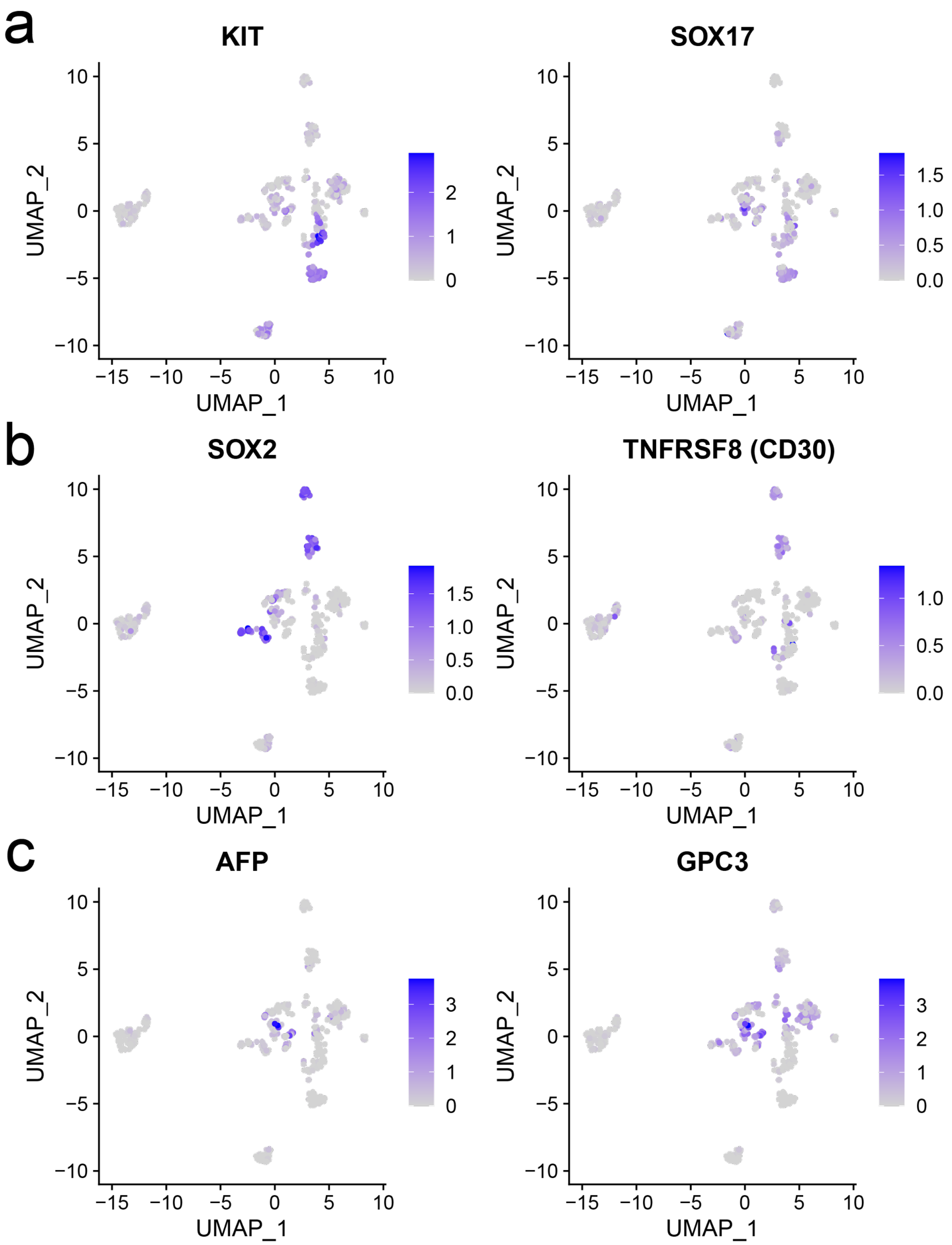

Supplementary Figure 14 | Normalised expression of marker genes that define GCT histologies. Marker genes were chosen for seminoma (a), embryonal carcinoma (b) and yolk sac tumour (c); the three histologies best characterised from previous studies [72 - 75]. The UMAP encompasses the transcriptomes of 416 microbiopsies.
