# Supplementary material for: Clonal diversification and histogenesis of malignant germ cell tumours
Source: Nat Commun. 2022 Aug 11;13:4272. doi: 10.1038/s41467-022-31375-4 (PMC9372159; doi:10.1038/s41467-022-31375-4)
Supplement: Supplementary file 3 — Description of Additional Supplementary Information [file 41467_2022_31375_MOESM3_ESM.pdf]

Description of Additional Supplementary Files

Supplementary Data 1. Overview of study cohort and histologies isolated for DNA and mRNA sequencing.

Supplementary Data 2. Overview of GCT whole genome sequencing data.

Supplementary Data 3. List of annotated substitutions and indels called across all GCT samples.

Supplementary Data 4. Copy number calls for all GCT samples that were estimated to have 5 or more reads per chromosome copy.

Supplementary Data 5. List of structural variants called across all GCT samples.

Supplementary Data 6. List of retrotransposition events called across all GCT samples.

Supplementary Data 7. Driver missense substitutions, their purity, and the copy number configuration at each locus.

Supplementary Data 8. Driver missense mutation detection across all GCT samples using CHASMplus.

Supplementary Data 9. Complete repertoire of SBS signatures extracted across all GCT samples.

Supplementary Data 10. Mutation clusters used to reconstruct the phylogenies shown in Supplementary Figure 6.

Supplementary Data 11. Proportion of invasive tumour substitutions found to lie in the trunk vs the overall burden.

Supplementary Data 12. Estimated pre-duplication substitution burden.

Supplementary Data 13. Estimation of WGD in mutation time per sample.

Supplementary Data 14. Overview of GCT mRNA sequencing data.

Supplementary Data 15. Differential gene expression between embryonal carcinoma and other GCT histologies microdissected, ranked by p value.

Supplementary Data 16. Gene expression enrichment by GCT tissue, binned per cytoband, relative to healthy adult seminiferous tubules.

Supplementary Data 17. Significantly overexpressed 12p genes, ranked by the number of invasive GCT tissues they are overexpressed in relative to normal testis.
