# Supplementary material for: Clonal diversification and histogenesis of malignant germ cell tumours
Source: Nat Commun. 2022 Aug 11;13:4272. doi: 10.1038/s41467-022-31375-4 (PMC9372159; doi:10.1038/s41467-022-31375-4)
Supplement: Supplementary file 5 — Description of Additional Supplementary Files [file 41467_2022_31375_MOESM5_ESM.docx]

Supplementary Figure 1. Histological images from each biopsy included in the microdissection cohort.

Supplementary Figure 2. Histograms of the cancer cell fraction for substitutions lying on the autosomal genome in each GCT sample.

Supplementary Figure 3. Aggregated copy number across the microdissection cohort compared to TCGA reference data.

Supplementary Figure 4. Trinucleotide context plots of all unique substitutions per GCT.

Supplementary Figure 5. Structural variant burden per invasive GCT.

Supplementary Figure 6. GCT phylogenies mapped back to histological sections.

Supplementary Figure 7. Overview of the mutationtimeR estimates for each copy gain called per GCT sample.

Supplementary Figure 8. Intra-tumoral yolk sac tumour transcriptional heterogeneity.

Supplementary Figure 9. The distribution of the mean log2 fold change in expression compared to normal testis tissues across 100,000 random subsets of genes from GCT genomic regions near baseline ploidy vs 12p genes.

Supplementary Figure 10. HDP signature extraction components.

Supplementary Figure 11. Distribution of the pre-QC total read depth for all microbiopsy transcriptomes.

Supplementary Figure 12. Distribution of the number of genes expressed to a depth of at least 5 reads for all pre-filtered transcriptomes.

Supplementary Figure 13. UMAP clustering of RNA microbiopsy data.

Supplementary Figure 14. Normalised expression of marker genes that define GCT histologies.
