# Supplementary material for: Clonal diversification and histogenesis of malignant germ cell tumours
Source: Nat Commun. 2022 Aug 11;13:4272. doi: 10.1038/s41467-022-31375-4 (PMC9372159; doi:10.1038/s41467-022-31375-4)
Supplement: Supplementary file 6 — Reporting Summary [file 41467_2022_31375_MOESM6_ESM.pdf]

nature portfolio

Corresponding author(s): S Behjati, R Rahbari

Last updated by author(s): Jun 7, 2022

Reporting Summary

Nature Portfolio wishes to improve the reproducibility of the work that we publish. This form provides structure for consistency and transparency in reporting. For further information on Nature Portfolio policies, see our Editorial Policies and the Editorial Policy Checklist.

Statistics

For all statistical analyses, confirm that the following items are present in the figure legend, table legend, main text, or Methods section.

n/a	Confirmed	
☐	☒	The exact sample size (*n*) for each experimental group/condition, given as a discrete number and unit of measurement
☐	☒	A statement on whether measurements were taken from distinct samples or whether the same sample was measured repeatedly
☐	☒	The statistical test(s) used AND whether they are one- or two-sided<br>*Only common tests should be described solely by name; describe more complex techniques in the Methods section.*
☐	☒	A description of all covariates tested
☐	☒	A description of any assumptions or corrections, such as tests of normality and adjustment for multiple comparisons
☐	☒	A full description of the statistical parameters including central tendency (e.g. means) or other basic estimates (e.g. regression coefficient) AND variation (e.g. standard deviation) or associated estimates of uncertainty (e.g. confidence intervals)
☐	☒	For null hypothesis testing, the test statistic (e.g. *F*, *t*, *r*) with confidence intervals, effect sizes, degrees of freedom and *P* value noted<br>*Give P values as exact values whenever suitable.*
☐	☒	For Bayesian analysis, information on the choice of priors and Markov chain Monte Carlo settings
☒	☐	For hierarchical and complex designs, identification of the appropriate level for tests and full reporting of outcomes
☐	☒	Estimates of effect sizes (e.g. Cohen's *d*, Pearson's *r*), indicating how they were calculated

Our web collection on statistics for biologists contains articles on many of the points above.

Software and code

Policy information about availability of computer code

Data collection	No software was used for data collection.
Data analysis	Bespoke R scripts used for analysis and visualisation in this study are available online from GitHub (https://github.com/trwo/GCT_diversification).<br><br>The additional packages and tools used in this manuscript are:<br>- Trim Galore (version 0.6.3)<br>- Spliced Transcripts Alignment to a Reference (STAR, version 2.5.2)<br>- featureCounts (version 1.5.1)<br>- Conpair (version 0.2)<br>- Mosdepth (version 0.2.3)<br>- CaVEMan (versions 1.13.14, 1.14.0, 1.14.1 and 1.15.1)<br>- Pindel (versions 3.2.0, 3.2.2 and 3.3.0)<br>- cgpBattenberg (version 3.5.3)<br>- DPClust (>5 samples, version 2.2.2; 5 or fewer samples, version 2.2.8)<br>- BRASS (versions 6.3.0 and 6.3.4)<br>- annotateBRASS (version 4)<br>- TraFic (version "TraFiC-mem")<br>- Hierarchical Dirichlet process (HDP, version 0.1.5)<br>- SigprofilerExtractor (version 1.0.15)<br>- CHASMplus (version 2.2.5)<br>- Shatterseek (version 0.4)<br>- mutationtimeR (version 1.00.2)

- edgeR (version 3.32.1)
- Limma (version 3.46.0)
- Seurat (version 4.0.1)

For manuscripts utilizing custom algorithms or software that are central to the research but not yet described in published literature, software must be made available to editors and reviewers. We strongly encourage code deposition in a community repository (e.g. GitHub). See the Nature Portfolio guidelines for submitting code & software for further information.

Data

Policy information about availability of data

All manuscripts must include a data availability statement. This statement should provide the following information, where applicable:

- Accession codes, unique identifiers, or web links for publicly available datasets
- A description of any restrictions on data availability
- For clinical datasets or third party data, please ensure that the statement adheres to our policy

The raw DNA data generated in this study have been deposited in the European Genome-Phenome Archive (EGA) under accession code EGAD00001007038 [https://wp.ega-archive.org/datasets/EGAD00001007038]. The raw RNA data generated in this study have been deposited in the EGA under accession code EGAD00001007037 [https://wp.ega-archive.org/datasets/EGAD00001007037]. Access to these datasets is restricted due to data privacy laws although access may be granted following an application to the Data Access Committee. The processed data are available in the Article and Supplementary Information. Where the underlying data is not contained within the Supplementary Information, Source data are provided with this paper.

Field-specific reporting

Please select the one below that is the best fit for your research. If you are not sure, read the appropriate sections before making your selection.

☒ Life sciences ☐ Behavioural & social sciences ☐ Ecological, evolutionary & environmental sciences

For a reference copy of the document with all sections, see nature.com/documents/nr-reporting-summary-flat.pdf

Life sciences study design

All studies must disclose on these points even when the disclosure is negative.

Sample size	No sample size calculation was performed. Sample size was determined by the availability of tissue and cost of the experiment. There is no accepted method by which one could power the phylogenetic analyses which represent a substantial portion of the experiment.
Data exclusions	DNA sequences were excluded where contamination from another individual was found during QC after sequencing. The Methods section outlines the criteria for excluding transcriptomic data.
Replication	All microdissection samples were analysed at once initially, without separate discovery and validation cohorts. For the distinct SBS signature patterns and differences in WGD timing we saw, an additional seven bulk yolk sac tumours were added and analysed together with the original microdissection cohort. These samples successfully confirmed these observations. Observations made regarding the novel mutational signature and discrepancies in structural variant burdens between pre- and postpubertal tumours were only made after the addition of these bulk samples.
Randomization	This doesn't apply to our study - no intervention/treatment was administered, nor were any a priori hypotheses specified. This is a descriptive analyses of a rare tumour type where subgrouping samples by their available patient characteristics, e.g. age and tumour histology, formed an integral part of the analysis.
Blinding	As mentioned above, this is a descriptive study that depends upon the available metadata to guide the analyses so it is difficult to say what aspect of this experiment could have been blinded. The variant calling and feature counting pipelines that were run did not depend on our subjective assessment.

Reporting for specific materials, systems and methods

We require information from authors about some types of materials, experimental systems and methods used in many studies. Here, indicate whether each material, system or method listed is relevant to your study. If you are not sure if a list item applies to your research, read the appropriate section before selecting a response.

Materials & experimental systems

n/a	Involved in the study
☐	☒ Antibodies
☒	☐ Eukaryotic cell lines
☒	☐ Palaeontology and archaeology
☒	☐ Animals and other organisms
☐	☒ Human research participants
☒	☐ Clinical data
☒	☐ Dual use research of concern

Methods

n/a	Involved in the study
☒	☐ ChIP-seq
☒	☐ Flow cytometry
☒	☐ MRI-based neuroimaging

Antibodies

Antibodies used	OCT3/4 (mouse monoclonal, Leica BOND PA0934, ready to use), AE1/3 (mouse monoclonal, Abcam ab27988, 1 in 20 dilution used), AFP (rabbit polyclonal, Dako A0008, 1 in 300 dilution used), GLYP3 (mouse monoclonal, Sigma-Aldrich 261M-96, 1 in 150 dilution), CD117 (rabbit monoclonal, Leica BOND PA0007, ready to use)
Validation	Each antibody was previously tested on control tissue to ensure staining specificity.

Human research participants

Policy information about studies involving human research participants

Population characteristics	Children and adults with malignant germ cell tumours. Metadata provided in Supplementary data 2.
Recruitment	Tissues were obtained either as archival specimens from biobanks or where clinical collaborators were able to identify suitable patients during their clinical practice over the course of 2019.
Ethics oversight	Ethical approval for this study was given by all the relevant local ethics committee (UK REC approval reference numbers 03/018, 08/h0405/22+5, 12/NE/0395, 16/EE/0394, 17/LO/1801,18/EM/0134 and 18/NW/0092). Informed, written consent was obtained from participants or their legal guardians. No compensation was provided to participants.

Note that full information on the approval of the study protocol must also be provided in the manuscript.
